# Attitudes of Men Who Have Sex With Men Toward HIV Functional Cure: Community-Based Study

**DOI:** 10.2196/79631

**Published:** 2026-02-19

**Authors:** Ngai Sze Wong, Sze Long Chung, Tsz Ho Kwan, Shui Shan Lee

**Affiliations:** 1 JC School of Public Health and Primary Care, The Chinese University of Hong Kong, Shatin, Hong Kong, China (Hong Kong); 2 S.H. Ho Research Centre for Infectious Diseases, The Chinese University of Hong Kong, Hong Kong, China (Hong Kong); 3 Stanley Ho Centre for Emerging Infectious Diseases, The Chinese University of Hong Kong, 2/F, Postgraduate Education Centre, Prince of Wales Hospital, Shatin, Hong Kong, China (Hong Kong), (852) 2252-8812

**Keywords:** HIV functional cure, HIV remission, men who have sex with men without HIV, attitudes, latent class analysis

## Abstract

**Background:**

Investigation of community awareness of HIV functional cure has often been restricted to people living with HIV. An understanding of the attitudes of men who have sex with men (MSM) without HIV is important, as it contributes to the supportiveness of curative treatment in the community.

**Objective:**

This study aimed to profile the attitudes toward HIV functional cure among MSM without HIV.

**Methods:**

This is a secondary analysis of data from a community-based survey on MSM 18 years or older in Hong Kong. MSM who self-reported being HIV negative were recruited for enrollment in the study. To understand the diversity of beliefs and their association with attitudes toward functional cure, attributes under the health belief model constructs were used for delineation of the study population by latent class analysis. Factors, including identified latent class analysis, associated with each outcome variable, including awareness, high anticipation, and trial participation willingness, were examined in bivariable and multivariable logistic regression models.

**Results:**

A total of 712 MSM were recruited between September 2021 and October 2022, a majority of whom were Chinese, single, and employed. In 541 (76%) MSM who self-reported not having tested positive for HIV, 43% (n=233) were aware of and 44% (n=236) held high anticipation toward functional cure, while 84% (n=452) indicated willingness to join clinical trials. Trial participation willingness and high anticipation were associated with indicators of higher risk sexual behavior. Four latent classes were identified. Compared with the reference group of class 4 (considered nontransmissibility as the main benefit, n=301, 56%), MSM in class 1 (selection of all perceived benefits, n=40, 7%) and class 2 (considered safety, trial duration, sites as most important, n=82, 15%) had significantly higher anticipation of functional cure, while those from class 2 were less likely to be aware of functional cure, and those from class 3 (no selection of reasons for joining trials, n=118, 22%) were less willing to participate in a trial.

**Conclusions:**

In Hong Kong, compared with MSM living with HIV, MSM without HIV had similar awareness and trial participation willingness but lower anticipation of functional cure, probably due to lower susceptibility to HIV infection. As community awareness and understanding of the functional cure concept take time to nourish, these have to be nurtured in advance while the development of the functional cure is in progress.

## Introduction

Antiretroviral therapy (ART) is a potent form of treatment that can suppress viral load and effectively restore immunity in people living with HIV. Life-long maintenance of ART is required, posing risks of adverse effects, poor adherence, and loss to follow-up. To overcome the shortcomings, new therapies are being trialed, which aim at maintaining viral load suppression without long-term treatment. Functional cure is a form of HIV therapy under development, which could maintain an undetectable viral load status in the absence of ART [1]. The outcome is also referred to as ART-free remission. Instead of a sterilizing cure, which aims at eradicating the virus, a functional cure aims to achieve a state of host-mediated viral replication control. Immune-based therapies involving broadly neutralizing antibodies and vaccines are being explored to achieve a functional cure [2]. In the past decade, novel interventions have been tested in clinical trials, showing the feasibility of HIV functional cure, while a sterilizing cure of HIV is still far from being achievable [3]. When functional cure–based therapies are successfully developed and implemented, people living with HIV could live without ART for an extended period or even for life. As people living with HIV on ART would stop therapy when functional cure is achieved, positive attitudes toward this new form of treatment are important. Previous studies [4-7] have shown the following perceived HIV cure outcomes in people living with HIV: free from HIV-related morbidities, no risk of transmitting HIV to sex partners, no requirement of HIV medication, no HIV in the body, and reduction of stigma. In general, people living with HIV showed positive attitudes toward functional cure [4,5].

While people living with HIV may face the new therapeutic challenge of functional cure, men who have sex with men (MSM) without HIV constitute the major stakeholders. Globally, the HIV prevalence is less than 10% in most MSM communities [8], implying that MSM without HIV account for a majority of the population. Failure of functional cure in people living with HIV while not on ART might suggest additional risk of virus transmission to MSM without HIV, after years of high ART coverage with the undetectable=untransmittable state. Perception of potential risk might discourage partners’ support for people living with HIV to start novel functional cure therapy. On the other hand, MSM with higher-risk sexual behavior are more likely to undergo repeat HIV testing and take pre-exposure prophylaxis (PrEP) [9,10], reflecting their higher perceived risk of HIV acquisition. In a qualitative study in the Netherlands, MSM without HIV considered reduction of HIV burden and increase of sexual freedom as the benefits of an HIV cure [11]. Their attitudes toward functional cure may be affected by their perceived susceptibility to HIV infection [11]. Their higher risk of HIV infection and the high subsequent likelihood of being introduced to functional cure make them a unique stakeholder group. Comparing the attitudes toward functional cure in different populations, a qualitative study in the Netherlands showed similar perceptions of the impact of HIV cure between people living with HIV and MSM without HIV [6]. However, in a qualitative study in China, people living with HIV ranked family, mental stress, and health as the top 3 themes of the potential benefits of HIV cure, while MSM without HIV ranked HIV and MSM stigma, sexual risk, and romantic relationship as the top themes [12]. A quantitative study suggested greater interest in functional cure research and higher frequency of information-seeking behavior in people living with HIV than people without HIV (including partners of people living with HIV and MSM) [13]. To our knowledge, no studies have quantitatively assessed the attitudes of MSM without HIV toward functional cure, except one that had not differentiated between partners of people living with HIV and MSM [13], and the limited number of studies in MSM without HIV were qualitative studies [6,11,12,14]. The attitudes toward HIV cure may be complex and varied among MSM without HIV in the community, but the potential patterns of attitudes could be explored and summarized systematically based on latent class analysis (LCA) of quantitative data. This study aimed to examine and profile the attitudes of HIV functional cure among MSM without HIV.

## Methods

### Study Area

Hong Kong is an international city in the Asia-Pacific region. The estimated HIV prevalence in the general population in Hong Kong in 2022 was 0.1%, but it was much higher at 6.6% in the MSM community [15]. MSM has been accounting for more than half of all new HIV diagnoses per year since 2012, the proportion of which has declined since 2018 [16].

### Study Population

This is a secondary analysis of data from a community-based online survey about HIV and sexually transmitted infections in MSM [17]. MSM aged 18 years or older who normally lived in Hong Kong were recruited through gay apps, online gay forums, and nongovernmental organizations. With e-consent, participants completed a self-administered online survey and received a HKD25 (US $1=HKD $7.8) catering voucher as an incentive for survey completion. The address and phone contact information provided for the delivery of incentives were used to check for duplication. The main survey items included sociodemographics, awareness, and attitudes toward HIV functional cure, sexual behavior, and practices of HIV prevention.

### Data Analysis

Characteristics of MSM who self-reported having tested negative for HIV (hereafter referred to as MSM without HIV) and those with unknown HIV status (hereafter referred to as unknown status) were compared in bivariable logistic regression. Data of MSM without HIV were then included in the main data analysis. To profile attitudes, the outcome variables were defined based on our previous study in people living with HIV [5], including dichotomized awareness of functional cure (at least knowing the idea), full score (10/10) on excitement level to reflect high anticipation, and willingness to participate in clinical trials of functional cure. Factors associated with each outcome variable were identified in bivariable and multivariable (stepwise backward) logistic regression models.

To account for the complexity of dimensions and the presence of a continuum of beliefs potentially associated with attitudes toward functional cure, LCA was applied to differentiate the patterns. LCA is a statistical model that classifies a population into unobserved groups that differ by attributes [18]. The attributes in LCA were selected based on the health belief model constructs, including perceived susceptibility, perceived severity, perceived benefits, perceived barriers and facilitators, and cues to action [19]. The variables were input in R version 4.2.2 (The R Foundation) using the poLCA package, with 4999 iterations. We tried 2 to 5 classes in LCA. Lower Bayesian information criterion (BIC), Akaike information criterion (AIC), chi-square statistics, higher entropy (at least 60%), and interpretability of identified classes were used to determine the number of classes in the model [20]. Multinomial logistic regression models were performed to compare the characteristics among the identified latent classes in SPSS. To account for the possible impact of MSM with unknown HIV status on functional cure attitudes and to test the robustness of the class structure, data of MSM without HIV and those with unknown status were input for sensitivity analysis in LCA. The associations between identified LCA classes and attitude variables (awareness, anticipation, and willingness to participate) were examined in bivariable and multivariable logistic regression. Complete case analyses were performed.

### Ethical Considerations

Ethical approval from the Joint Chinese University of Hong Kong-New Territories East Cluster Clinical Research Ethics Committee was obtained (approval CREC2020.436). Electronic consent was obtained before the start of the online survey. Participants received a catering voucher with a face value of HK $25 (~US $3.20) after submission of the online survey. All data are password protected and only accessible by project investigators and designated research staff.

## Results

### Characteristics of MSM Without HIV and With Unknown HIV Status

Between September 2021 and October 2022, 541 (78%) MSM without HIV and 150 (22%) MSM with unknown status were recruited. The median age of MSM without HIV was 30 (IQR 26‐35) years, while the median age of MSM with unknown HIV status was 30.5 (IQR 26‐35) years. A majority of the recruited MSM were Chinese, single, employed, and had attained secondary school education level or above (Table 1). Compared with MSM without HIV, MSM with unknown HIV status were more likely to have a monthly income below HKD $50,000. They were less likely to be PrEP experienced (odds ratio [OR] 0.36, 95% CI 0.21‐0.63), to have engaged in chemsex (OR 0.50, 95% CI 0.27‐0.91), and to have engaged in group sex (OR 0.50, 95% CI 0.35‐0.72), although chemsex engagement in the past 6 months was not significantly different. MSM with unknown HIV status were also less sexually active (OR 0.27, 95% CI 0.15‐0.50) and less likely to have participated in sexual partner networking events through gay apps (OR 0.66, 95% CI 0.45‐0.96) and social media (OR 0.68, 95% CI 0.47‐0.99) in the past 6 months.

**Table 1.** Characteristics of men who have sex with men self-reported without HIV or unknown HIV status.

	Latest HIV test results, n (%)				Bivariable logistic regression, OR^a^ (95% CI)	
	Negative (n=541)		Do not know (n=150)			
Sociodemographics						
Age group (y)						
18‐29	245 (45)		70 (47)		1.10 (0.64‐1.91)	
30‐39	215 (40)		59 (39)		1.06 (0.60‐1.85)	
≥40	81 (15)		21 (14)		Ref^b^	
Ethnicity (n=689)						
Non-Chinese	7 (1)		1 (1)		Ref	
Chinese	533 (99)		148 (99)		1.94 (0.24‐15.92)	
Marital status						
Married or civil union	17 (3)		4 (3)		Ref	
Single	524 (97)		146 (97)		1.18 (0.39‐3.57)	
In employment						
No	84 (16)		29 (19)		Ref	
Yes	457 (84)		121 (81)		0.77 (0.48‐1.22)	
Education level (n=690)						
Secondary	71 (13)		27 (18)		Ref	
Above secondary	470 (87)		122 (82)		0.68 (0.42‐1.11)	
Monthly income (n=62; HK $^c^)						
<15,000	118 (24)		32 (25)		3.25^f^ (1.30‐8.16)	
15,000‐30,000	223 (45)		66 (51)		3.55^f^ (1.48‐8.54)	
30,001‐50,000	83 (17)		26 (20)		3.76^f^ (1.47‐9.64)	
≥50,000	72 (15)		6 (5)		Ref	
HIV/STI^d^ prevention						
PrEP^e^ experience (n=682)						
Never	399 (74)		125 (89)		Ref	
Ever	142 (26)		16 (11)		0.36^f^ (0.21‐0.63)	
Sexual behavior						
Ever chemsex engagement						
No	454 (84)		137 (91)		Ref	
Yes	87 (16)		13 (9)		0.50^f^ (0.27‐0.91)	
Every group sex engagement						
No	197 (36)		80 (53)		Ref	
Yes	344 (64)		70 (47)		0.50^f^ (0.35‐0.72)	
STI diagnosis in the past 1 y (n=690)						
No	468 (87)		139 (93)		Ref	
Yes	72 (13)		11 (7)		0.51^f^ (0.27‐0.997)	
History of sex in the past 6 mo						
No	24 (4)		22 (15)		Ref	
Yes	517 (96)		128 (85)		0.27^f^ (0.15‐0.50)	
Chemsex engagement in the past 6 mo						
No	499 (92)		143 (95)		Ref	
Yes	42 (8)		7 (5)		0.58 (0.26‐1.32)	
Sex partner networking event in the past 6 mo (not mutually exclusive)						
Gay apps	392 (72)		95 (63)		0.66^f^ (0.45‐0.96)	
Social media	256 (47)		57 (38)		0.68^f^ (0.47‐0.99)	
Sauna	73 (13)		18 (12)		0.87 (0.50‐1.52)	
Gay bars	20 (4)		8 (5)		1.47 (0.63‐3.40)	

a ORw: odds ratio.

b Ref: reference.

c US $1=HK $7.8.

d STI: sexually transmitted infection.

e PrEP: pre-exposure prophylaxis.

f *P*<.05.

### Pattern of Beliefs Potentially Associated With Attitudes Toward Functional Cure in LCA

Among MSM without HIV, 4 latent classes were identified by LCA (BIC=9149; AIC=8878; and entropy=71.5%; Multimedia Appendix 1). A majority of *class 1 (high anticipation and willingness*) MSM (n=40, 7%) selected almost all perceived benefits of functional cure (Table 2). More than 90% of *class 2 (high anticipation but low awareness*) MSM (n=82, 15%) perceived safety and the duration of clinical trial as influencing factors for participation and considered the advice of clinical staff and studies by reputable research organizations important in encouraging them to participate. Less than 60% of *class 3 (moderate anticipation and low willingness*) MSM (n=118, 22%) perceived that these were benefits of functional cure. Only a small proportion of them selected factors that might affect their participation in functional cure trials, of which one-third selected no participation for any reason. In *class 4 (moderate anticipation and high willingness*; n=301, 56%), more than half (75%) of the MSM perceived the benefit of functional cure as the state of nontransmissibility.

**Table 2.** Pattern of beliefs potentially associated with attitudes toward functional cure among men who have sex with men (MSM) self-reporting HIV negative test results (n=541).

LCA^a^ attributes	MSM, n (%)	Latent classes^b^			
		Class 1—high anticipation and willingness^c^, n (%)	Class 2—high anticipation but low awareness^d^, n (%)	Class 3—moderate anticipation and low willingness^e^, n (%)	Class 4—moderate anticipation and high willingness^f^, n (%)
Perceived susceptibility					
Sex partner living with HIV					
None	324 (60)	18 (45)	51 (62)	70 (59)	185 (61)
At least one	41 (8)	4 (10)	5 (6)	14 (12)	18 (6)
Not sure	176 (33)	18 (45)	26 (32)	34 (29)	98 (33)
Perceived severity (indirectly from the meaning of functional cure)					
Risk of progressing to AIDS or developing other complications	251 (46)	37 (93)	37 (45)	30 (25)	147 (49)
Impairment of immune function	356 (66)	40 (100)	49 (60)	73 (62)	194 (64)
Perceived benefits					
No longer needing to take life-long medicine	287 (53)	40 (100)	44 (54)	69 (58)	134 (45)
No transmission of HIV to others	379 (70)	40 (100)	55 (67)	59 (50)	225 (75)
No more positive markers for infection	177 (33)	39 (98)	44 (54)	21 (18)	73 (24)
Feel like immune to HIV, never be infected with HIV anymore	90 (17)	27 (68)	5 (6)	29 (25)	29 (10)
No longer have to seek medical help	135 (25)	40 (100)	3 (4)	25 (21)	67 (22)
Perceived barriers and facilitators					
Safety of the treatment	419 (77)	36 (90)	77 (94)	23 (19)	283 (94)
Duration of the study	289 (53)	27 (68)	75 (91)	12 (10)	175 (58)
Support from friends and family	29 (5)	4 (10)	11 (13)	5 (4)	9 (3)
The need to stop other medicine	125 (23)	14 (35)	41 (50)	0 (0)	70 (23)
Cues to action					
Advice of clinical staff	196 (36)	24 (60)	78 (95)	29 (25)	65 (22)
Reputation of research organization	229 (42)	26 (65)	81 (99)	21 (18)	101 (34)

a LCA: latent class analysis.

b Bayesian information criterion 9149; Akaike information criterion 8878; relative entropy 71.5%.

c Class 1: selection of all perceived benefits (n=40, 7%).

d Class 2: considered safety, trial duration, and sites as most important (n=82, 15%).

e Class 3: no selection of reasons for joining trials (n=118, 22%).

f Class 4: considered nontransmissibility as the main benefit (n=301, 56%).

When comparing the characteristics among MSM in the 4 classes, class 4 MSM were more likely to be single (OR 3.10, 95% CI 1.02‐9.43) than class 3 MSM (Table 3). Moreover, class 2 MSM were more likely to be PrEP experienced (OR 2.14, 95% CI 1.11‐4.12) compared with class 3 MSM. No other factors were significantly associated, although the proportion of MSM aged 18 to 29 years in class 2 was high (44/82, 54%) and the proportion of sexually transmitted infection diagnosis in the past 1 year in class 1 was high at 20% (8/40).

**Table 3.** Factors associated with the 4 identified classes in latent class analysis.

	Class 1 (n=40), n (%)	Class 2 (n=82), n (%)	Class 3 (n=118), n (%)	Class 4 (n=301), n (%)	Multinomial logistic regression (reference: class 3^a^)		
					Class 1^b^, OR^c^ (95% CI)	Class 2^d^, OR (95% CI)	Class 4^e^, OR (95% CI)
Sociodemographics							
Age group (y)							
18‐29	17 (43)	44 (54)	55 (47)	129 (43)	0.54 (0.19‐1.51)	1.40 (0.54‐3.64)	0.64 (0.33‐1.26)
30‐39	15 (38)	30 (37)	49 (42)	121 (40)	0.54 (0.19‐1.52)	1.07 (0.4‐2.86)	0.68 (0.34‐1.34)
≥40	8 (20)	8 (10)	14 (12)	51 (17)	Ref^f^	Ref	Ref
Chinese (ref: non-Chinese; n=540)	39 (98)	81 (99)	117 (99)	296 (99)	0.33 (0.02‐5.46)	0.69 (0.04‐11.23)	0.63 (0.07‐5.72)
Being single (ref: married or civil union)	38 (95)	80 (98)	111 (94)	295 (98)	1.20 (0.24‐6.02)	2.52 (0.51‐12.46)	3.10^g^ (1.02‐9.43)
In employment (ref: no)	33 (83)	70 (85)	97 (82)	257 (85)	1.02 (0.40‐2.62)	1.26 (0.58‐2.74)	1.26 (0.72‐2.24)
Above secondary education level (ref: secondary)	33 (83)	73 (89)	104 (88)	260 (86)	0.63 (0.24‐1.70)	1.09 (0.45‐2.66)	0.85 (0.45‐1.63)
Monthly income (HK $^h^; n=496)							
<$15,000	6 (17)	17 (23)	28 (27)	67 (24)	0.64 (0.13‐3.11)	0.42 (0.15‐1.19)	0.46 (0.20‐1.06)
$15,000‐$30,000	15 (42)	31 (42)	51 (49)	126 (45)	0.88 (0.21‐3.68)	0.42 (0.16‐1.10)	0.47 (0.22‐1.04)
$30,001‐$50,000	12 (33)	13 (18)	17 (16)	41 (15)	2.12 (0.47‐9.50)	0.53 (0.17‐1.61)	0.46 (0.19‐1.15)
≥$50,000	3 (8)	13 (18)	9 (9)	47 (17)	Ref	Ref	Ref
Sexual behavior and HIV prevention							
Ever group sex	24 (60)	53 (65)	69 (58)	198 (66)	1.07 (0.51‐2.21)	1.30 (0.73‐2.32)	1.37 (0.88‐2.11)
Chemsex engagement in the past 6 mo	2 (5)	8 (10)	8 (7)	24 (8)	0.72 (0.15‐3.56)	1.49 (0.53‐4.14)	1.19 (0.52‐2.73)
STI^i^ diagnosis in the past 1 y	8 (20)	11 (14)	15 (13)	38 (13)	1.72 (0.67‐4.42)	1.08 (0.47‐2.49)	0.99 (0.52‐1.88)
PrEP^j^ experience	12 (30)	27 (33)	22 (19)	81 (27)	1.87 (0.82‐4.24)	2.14^g^ (1.11‐4.12)	1.61 (0.95‐2.73)
HIV testing in the past 1 y	33 (83)	68 (83)	98 (83)	250 (83)	0.96 (0.37‐2.48)	0.99 (0.47‐2.10)	1.00 (0.57‐1.76)

a Class 3: no selection of reasons for joining trials.

b Class 1: selection of all perceived benefits.

c OR: odds ratio.

d Class 2: considered safety, trial duration, and sites as most important.

e Class 4: considered nontransmissibility as the main benefit.

f Ref: reference.

g *P*<.05.

h US $1=HK $7.8.

i STI: sexually transmitted infection.

j PrEP: pre-exposure prophylaxis.

In the sensitivity analysis including data of MSM with unknown HIV status , 4 latent classes could be identified (BIC=11,485; AIC=11200; and entropy=69.4%; Multimedia Appendix 2). The distribution of attributes in the identified latent classes was similar to that of MSM without HIV, within a 10% difference, except for a lower proportion of class 1 and class 3 MSM who considered treatment safety and class 1 MSM who considered trial duration as the potential facilitators and barriers to participation in the sensitivity analysis (Multimedia Appendix 3).

### Factors Associated With Awareness and High Anticipations of Functional Cure and Trial Participation Willingness

Among MSM without HIV, 43% (233/541) were aware of functional cure, with 84% (452/541) indicating willingness to join related clinical trials, while 44% (236/541) gave a full score for excitement level. In bivariable logistic regression, LCA classes were the only factor significantly associated with each of these outcomes (Table 4).

**Table 4.** Factors associated with awareness and high anticipations of functional cure and trial participation willingness in men who have sex with men self-reported without HIV (n=541).

	Aware of functional cure, OR^a^ (95% CI)		High anticipations (full score excitement), OR (95% CI)		Willing to participate, OR (95% CI)	
Sociodemographics						
Age group (y)						
18‐29	1.43 (0.85‐2.40)		0.62 (0.37‐1.02)		0.68 (0.32‐1.43)	
30‐39	1.25 (0.74‐2.11)		0.85 (0.51‐1.41)		0.68 (0.32‐1.44)	
≥40	Ref^b^		Ref		Ref	
Chinese (ref: non-Chinese; n=540)	1.91 (0.37‐9.94)		0.31 (0.06‐1.59)		—^c^	
Being single (ref: married or civil union)	0.52 (0.19‐1.38)		0.53 (0.20‐1.42)		1.59 (0.51‐4.99)	
In employment (ref: no)	0.54^d^ (0.34‐0.86)		0.93 (0.58‐1.48)		0.92 (0.48‐1.74)	
Above secondary education level (ref: secondary)	0.97 (0.59‐1.61)		1.07 (0.64‐1.77)		1.16 (0.61‐2.23)	
Monthly income (HK $^e^; n=496)						
<$15,000	1.52 (0.84‐2.76)		1.17 (0.64‐2.15)		0.84 (0.37‐1.93)	
$15,000‐30,000	1.02 (0.59‐1.77)		1.60 (0.93‐2.77)		0.70 (0.33‐1.47)	
$30,001‐50,000	1.33 (0.70‐2.52)		1.36 (0.71‐2.59)		1.18 (0.46‐3.01)	
>$50,000	Ref		Ref		Ref	
Health status and sexual behavior						
HIV testing 1 y (ref: no)	1.28 (0.81‐2.04)		1.18 (0.75‐1.87)		1.19 (0.66‐2.13)	
STI^f^ testing 1 y (ref: no)	1.18 (0.84‐1.67)		1.03 (0.73‐1.44)		0.88 (0.56‐1.40)	
History of STI diagnosis in the past 1 y (ref: no)	1.47 (0.89‐2.41)		1.52 (0.93‐2.51)		5.18^d^ (1.59‐16.84)	
PrEP^g^-experienced (ref: PrEP naïve; n=527)	0.96 (0.65‐1.41)		1.31 (0.89‐1.93)		3.26^d^ (1.64‐6.49)	
Sex partner living with HIV						
None	1.69^d^ (1.15‐2.47)		0.82 (0.57‐1.19)		0.70 (0.42‐1.16)	
At least 1 person living with HIV	2.39^d^ (1.17‐4.86)		2.36^d^ (1.13‐4.91)		2.98 (0.67‐13.16)	
All were people living with HIV	0.97 (0.09‐10.88)		2.46 (0.22‐27.58)		—	
Not sure	Ref		Ref		Ref	
Ever chemsex engagement (ref: never)	0.87 (0.55‐1.39)		0.90 (0.56‐1.43)		3.72^d^ (1.46‐9.47)	
Ever group sex (ref: never)	1.10 (0.77‐1.56)		1.21 (0.85‐1.73)		2.52^d^ (1.59‐3.99)	
In the past 6 mo						
Sought new sex partners	0.88 (0.51‐1.52)		0.84 (0.49‐1.44)		1.34 (0.68‐2.65)	
Sex partner networking events (not mutually exclusive)						
Gay apps	1.05 (0.71‐1.53)		0.96 (0.66‐1.41)		1.10 (0.67‐1.82)	
Social media	0.93 (0.66‐1.31)		0.66^d^ (0.47‐0.93)		0.95 (0.60‐1.50)	
Sauna	0.91 (0.55‐1.50)		1.91^d^ (1.16‐3.15)		2.00 (0.89‐4.52)	
Gay bars	2.54 (0.997‐6.47)		1.06 (0.43‐2.60)		1.12 (0.32‐3.91)	
History of sex (ref: no)	0.89 (0.39‐2.02)		1.09 (0.47‐2.49)		1.74 (0.67‐4.52)	
Number of nonregular sex partners (n=514)						
0	0.98 (0.63‐1.52)		1.24 (0.80‐1.91)		0.40^d^ (0.22‐0.75)	
1‐4	1.03 (0.68‐1.55)		1.05 (0.69‐1.59)		0.57 (0.30‐1.05)	
≥5	Ref		Ref		Ref	
Number of regular sex partners (n=514)						
0	0.96 (0.61‐1.50)		0.76 (0.49‐1.19)		0.40^d^ (0.22‐0.74)	
1	0.99 (0.66‐1.48)		0.59^d^ (0.39‐0.88)		0.48^d^ (0.27‐0.86)	
≥2	Ref		Ref		Ref	
At least one commercial sex partner (ref: zero; n=514)	0.96 (0.49‐1.88)		1.31 (0.68‐2.54)		0.43^d^ (0.20‐0.91)	
Always condom usage (ref: inconsistent; n=506)	0.47^d^ (0.31‐0.71)		1.12 (0.75‐1.65)		0.79 (0.47‐1.32)	
Chemsex engagement (ref: no)	0.99 (0.52‐1.87)		1.07 (0.57‐2.02)		8.78^d^ (1.19‐64.67)	
Suspected to have STI (ref: no; n=523)	0.84 (0.58‐1.24)		1.30 (0.89‐1.89)		1.33 (0.78‐2.27)	
LCA^h^ classes ^i^						
Class 1^j^—high anticipation and willingness	0.87 (0.45‐1.70)		3.57^d^ (1.75‐7.29)		1.04 (0.39‐2.82)	
Class 2^k^—high anticipation but low awareness	0.49^d^ (0.29‐0.83)		1.95^d^ (1.19‐3.20)		1.07 (0.51‐2.25)	
Class 3^l^—moderate anticipation and low willingness	1.00 (0.65‐1.53)		0.88 (0.56‐1.36)		0.35^d^ (0.21‐0.59)	
Class 4^m^—moderate anticipation and high willingness	Ref		Ref		Ref	

a OR: odds ratio.

b Ref: reference.

c Not applicable.

d *P*<.05.

e US $1=HK $7.8.

f STI: sexually transmitted infection.

g PrEP: pre-exposure prophylaxis.

h LCA: latent class analysis.

i Identified latent classes in Table 2.

j Class 1: selection of all perceived benefits.

k Class 2: considered safety, trial duration, and sites as most important.

l Class 3: no selection of reasons for not joining trials.

m Class 4: considered nontransmissibility as the main benefit.

In the multivariable logistic regression model, being unemployed (adjusted OR [aOR] 2.00, 95% CI 1.20‐3.33), inconsistent condom use in the past 6 months (aOR 1.97, 95% CI 1.28‐3.04), and reporting zero partner living with HIV (with “not sure about partners’ HIV status” as reference group; aOR 1.72, 95% CI 1.14‐2.60) were positively associated with awareness. In the same model, compared with class 4 MSM, class 2 MSM (aOR 0.52, 95% CI 0.30‐0.90) were less likely to be aware of functional cure. In a multivariable logistic regression model for high anticipation, having at least one partner living with HIV (with “not sure about HIV status” as the reference group; aOR 2.58, 95% CI 1.16‐5.75), having sex partner networking events in saunas (aOR 1.76, 95% CI 1.03‐3.02) but not through social media (with “social media” as the reference group; aOR 1.54, 95% CI 1.06‐2.23), and having at least 5 regular sex partners (1‐4 as the reference group; aOR 1.81, 95% CI 1.18‐2.79) in the past 6 months, and belonging to class 1 (aOR 3.41, 95% CI 1.63‐7.15) and class 2 (aOR 1.95, 95% CI 1.16‐3.26) were significant factors. In the model for willingness to participate in clinical trials, only group sex engagement in the past 6 months (aOR 1.81, 95% CI 1.09‐3.03) and belonging to class 3 (with class 4 as the reference group; aOR 0.32, 95% CI 0.18‐0.57) were significantly associated factors.

## Discussion

### Principal Findings

Community support is important for the successful implementation of HIV treatment among people living with HIV. This is particularly crucial for new forms of therapy such as functional cure–based treatment, the implementation of which is anticipated as the next milestone in advanced HIV therapeutics. In this study, we profiled attitudes toward functional cure in MSM without HIV in Hong Kong, a city where the coverage of ART is high among people living with HIV[21]. Compared with a previous study in MSM living with HIV in Hong Kong, a similar proportion of our study participants were aware of functional cure (42% vs 43% in this study), and most were willing to join a functional cure trial (93% vs 92% in this study), but a smaller proportion scored full excitement level (59% vs 44% in this study) [5].

In MSM without HIV, high anticipation of functional cure and willingness to participate in a functional cure trial were associated with indicators of recent practice of higher risk sexual behavior. Participants in a previous qualitative study expressed more liberal attitudes toward sexual behavior if an HIV cure can be achieved [10]. The practice of high-risk sexual behavior may result in a lower risk of acquiring HIV if most of the people living with HIV in the community achieve functional cure. This explains why they were supportive and willing to contribute to the development of an HIV functional cure despite their HIV negative status. On the other hand, a PrEP awareness study in China suggested that young MSM who had sex partner networking events in physical venues were more likely to be aware of and willing to take PrEP than those who met partners through the internet, probably due to insufficient online promotion [22]. Our study findings showed no significant differences in willingness to join HIV cure trials by types of sex partner networking events. However, the positive association of events in gay saunas and the negative association of events through social media with high anticipation of cure were in line with the observations from the previous study [22]. When there is trial recruitment, messaging at physical venues, such as gay saunas, might enable potential participants with high anticipation to be enrolled. Consistent with the previous study, those with at least one sex partner living with HIV had more positive attitudes or were more supportive [23]. On the other hand, engagement with existing biomedical prevention measures might affect attitudes toward functional cure. In our study, PrEP-experienced MSM showed higher willingness to participate in a cure trial, who might be more familiar with and more open to novel biomedical interventions.

In this study, we delineated MSM without HIV into 4 latent classes, of which class 2 had significantly lower awareness but higher anticipation than class 4. This was consistent with the findings from a study of MSM living with HIV, suggesting a significant negative association between anticipation and awareness [5]. Promotion of the functional cure concept and related knowledge would be needed to ground the expectations of class 2 MSM. Distinguishing the concept of functional cure from that of sterilizing cure to avoid misconception is important in communicating with potential participants. MSM in class 3 (n=118, 22%) and class 4 (n=301, 56%) had similar levels of awareness and moderate anticipation of functional cure, but class 3 had significantly lower willingness to participate. Class 3 MSM could be similar to participants in a previous qualitative study, who had lower perceived susceptibility to HIV. They might not consider HIV functional cure as a therapy highly relevant to or affecting them, reflecting a perceived distance from personal benefits [11]. Community education focusing on personal relevance and then extending to the broader MSM community might be useful for targeting class 3 MSM. On the other hand, three-quarters of MSM in class 4 perceived the nontransmissibility of HIV as the main benefit of functional cure, which was consistent with the perceived benefits of reduced HIV burden and more sexual freedom shown in the previous qualitative study [11].

As a novel biomedical intervention, both the community and people living with HIV need time to comprehend the underlying concept of HIV functional cure before a future new treatment strategy is implemented. The lesson derived from the undetectable=untransmittable regarding HIV therapy is that increasing awareness [24] is necessary but not sufficient. It would be important to reduce potential misunderstanding of functional cure in the community, notably MSM without HIV but at higher risk of infection. Even among people living with HIV, knowledge of HIV cure–related research was limited, and their understanding of functional cure needs to be further enhanced [25]. In parallel with progress in the scientific development of functional cure, promotion of community awareness among people at higher risk of infection is much needed.

This study has a few limitations. We applied the same survey items from the previous local study on functional cure for people living with HIV [5], which may not be fully applicable for people without HIV, as they did not have the same concern about treatment interruption nor the perceived stigma associated with HIV status. The responses to hypothetical questions may not reflect the actual thoughts and willingness, as suggested in the previous study [26]. Misclassification in LCA might bias the findings toward the null hypothesis. Compared with the previous local community-based survey [15], our study population, regardless of HIV status (n=712) [17], was younger, with 85% (n=607) aged 18 to 39 years (vs 70% in the previous study), but had a similar proportion of Chinese ethnicity and ever having HIV testing coverage. However, we acknowledge the cultural specificity of Hong Kong, the findings and implications of which might not be extrapolated to other places. As with other surveys, recall bias in respondents’ sexual behavior and potential bias in response quality caused by incentives could exist. Finally, there is no validation of participants’ self-reported HIV status. Regardless of the last HIV testing time, participants’ responses on their beliefs and attitudes toward functional cure should remain valid, as they were provided under the perception of being uninfected.

### Conclusions

MSM without HIV are a key early audience for functional cure communication and are stakeholders of functional cure. While MSM without HIV are not direct recipients of functional cure therapies, their awareness and willingness to join a functional cure trial were similar to MSM living with HIV in a previous local study, but with lower anticipation. Overall, 4 latent classes of MSM without HIV can be differentiated, which varied by their perceived susceptibility to infection, benefits, barriers and facilitators, and cues to action. As community awareness and understanding of the functional cure concept take time to nourish, these have to be nurtured in parallel with the scientific progress of functional cure development.

## Supplementary material

10.2196/79631 Multimedia Appendix 1 Comparison on the performance of latent class analysis, 4999 iterations, for men who have sex with men without HIV (n=541).

10.2196/79631 Multimedia Appendix 2 Comparison of the performance of latent class analysis, 4999 iterations, for men who have sex with men without HIV or with unknown HIV status (N=684).

10.2196/79631 Multimedia Appendix 3 Latent pattern of attitude toward HIV functional cure among men who have sex with men self-reported without HIV or unknown HIV status (N=684).

## Abbreviations

AIC: Akaike information criterion
aOR: adjusted odds ratio
ART: antiretroviral therapy
BIC: Bayesian information criterion
LCA: latent class analysis
MSM: men who have sex with men
OR: odds ratio
PrEP: pre-exposure prophylaxis
