# Supplementary material for: Attitudes of Men Who Have Sex With Men Toward HIV Functional Cure: Community-Based Study
Source: JMIR Form Res. 2026 Feb 19;10:e79631. doi: 10.2196/79631 (PMC12919903; doi:10.2196/79631)
Supplement: Multimedia Appendix 1 [file formative-v10-e79631-s001.docx]

**Supplementary Table 1. Comparison on the performance of latent class analysis, 4999 iterations, for MSM without HIV, N= 541**

| **No. of classes** | **BIC** | **AIC** | **X^2^** | **Entropy** | **Predicted class memberships** | | | | |
| --- | --- | --- | --- | --- | --- | --- | --- | --- | --- |
|  |  |  |  |  | **Class 1** | **Class 2** | **Class 3** | **Class 4** | **Class 5** |
| 2 | 9150 | 9017 | 41985 | 53.1% | 35.9% | 64.1% |  |  |  |
| 3 | 9189 | 8987 | 33263 | 56.8% | 16.3% | 62.1% | 21.6% |  |  |
| 4 | 9149 | 8878 | 32883 | 71.5% | 7.4% | 15.2% | 21.8% | 55.6% |  |
| 5 | 9202 | 8863 | 39776 | 76.9% | 49.9% | 15.9% | 7.0% | 3.5% | 23.7% |

AIC - Akaike information criterion; BIC – Bayesian information criterion; X2 Chi-square goodness of fit;

Model with 4 classes was selected with the lowest AIC and X2 value, and the highest entropy.
