# Supplementary material for: Attitudes of Men Who Have Sex With Men Toward HIV Functional Cure: Community-Based Study
Source: JMIR Form Res. 2026 Feb 19;10:e79631. doi: 10.2196/79631 (PMC12919903; doi:10.2196/79631)
Supplement: Multimedia Appendix 2 [file formative-v10-e79631-s002.docx]

**Supplementary Table 2. Comparison on the performance of latent class analysis, 4999 iterations, for MSM without HIV or with unknown HIV status, N=684**

| **No. of classes** | **BIC** | **AIC** | **X^2^** | **Entropy** | **Predicted class memberships** | | | | |
| --- | --- | --- | --- | --- | --- | --- | --- | --- | --- |
|  |  |  |  |  | **Class 1** | **Class 2** | **Class 3** | **Class 4** | **Class 5** |
| 2 | 11568 | 11428 | 34687 | 53.3% | 44.4% | 55.6% |  |  |  |
| 3 | 11608 | 11395 | 29157 | 60.6% | 9.9% | 41.1% | 49.0% |  |  |
| 4 | 11485 | 11200 | 35053 | 69.4% | 7.8% | 13.2% | 22.7% | 56.4% |  |
| 5 | 11558 | 11201 | 22440 | 78.2% | 20.5% | 7.6% | 20.6% | 7.0% | 44.3% |

AIC - Akaike information criterion; BIC - Bayesian information criterion; X^2^ Chi-square goodness of fit

Model with 2 classes was selected with the lowest BIC and the highest entropy.
