# Supplementary material for: Attitudes of Men Who Have Sex With Men Toward HIV Functional Cure: Community-Based Study
Source: JMIR Form Res. 2026 Feb 19;10:e79631. doi: 10.2196/79631 (PMC12919903; doi:10.2196/79631)
Supplement: Multimedia Appendix 3 [file formative-v10-e79631-s003.docx]

**Supplementary Table 3. Latent pattern of attitude towards HIV functional cure among MSM self-reported without HIV or unknown HIV status (N=684)**

|  |  | **Latent classes (BIC =11485, AIC=11200, relative entropy=69.4%)** | | | |
| --- | --- | --- | --- | --- | --- |
|  |  | **Class 1** | **Class 2** | **Class 3** | **Class 4** |
|  | % of MSM | n=53 | n=90 | n=155 | n=386 |
| Probability of membership |  | 8% | 13% | 23% | 56% |
| **LCA attributes** |  |  |  |  |  |
| **Perceived susceptibility** |  |  |  |  |  |
| Sex partner living with HIV |  |  |  |  |  |
| none | 61% | 53% | 60% | 60% | 62% |
| not sure | 33% | 42% | 34% | 33% | 31% |
| at least one | 7% | 6% | 6% | 7% | 7% |
| **Perceived severity (indirectly from meaning of functional cure)** |  |  |  |  |  |
| risk of progressing to AIDS or developing other complications | 45% | 96% | 47% | 29% | 44% |
| impairment of immune function | 65% | 100% | 62% | 55% | 65% |
| **Perceived benefits** |  |  |  |  |  |
| No longer needing to take life-long medicine | 53% | 100% | 59% | 52% | 45% |
| No transmission of HIV to others | 68% | 100% | 61% | 45% | 75% |
| No more positive markers for infection | 32% | 96% | 58% | 17% | 24% |
| Feel like immune to HIV, never be infected with HIV anymore | 17% | 77% | 7% | 14% | 12% |
| No longer have to seek medical help | 27% | 100% | 6% | 28% | 22% |
| **Perceived barriers and facilitators** |  |  |  |  |  |
| Safety of the treatment | 74% | 77% | 92% | 8% | 95% |
| Duration of the study | 51% | 57% | 98% | 11% | 55% |
| Support from friends and family | 6% | 6% | 16% | 5% | 4% |
| The need to stop other medicine | 21% | 26% | 58% | 2% | 20% |
| **Cues to action** |  |  |  |  |  |
| Advice of clinical staff | 35% | 53% | 93% | 18% | 26% |
| Reputation of research organisation | 41% | 58% | 98% | 14% | 36% |

LCA – latent class analysis
